# Introducing responsible innovation in health: a policy-oriented framework

**DOI:** 10.1186/s12961-018-0362-5

**Published:** 2018-09-10

**Authors:** Hudson Pacifico Silva, Pascale Lehoux, Fiona Alice Miller, Jean-Louis Denis

**Affiliations:** 1 Institute of Public Health Research of University of Montreal (IRSPUM), Montreal, Canada; 2 Department of Health Management, Evaluation and Policy, University of Montreal, P.O. Box 6128, Branch Centre-ville, Montreal, Quebec H3C 3J7 Canada; 3 University of Montreal Chair on Responsible Innovation in Health, Institute of Public Health Research of University of Montreal (IRSPUM), Research Center of the University of Montreal Health Center (CRCHUM), P.O. Box 6128, Branch Centre-ville, Montreal, Quebec H3C 3J7 Canada; 4 Institute of Health Policy, Management and Evaluation, University of Toronto, Toronto, Canada; 5 Department of Health Management, Evaluation and Policy, School of Public Health, University of Montreal, Montreal, Canada; 6 Canada Research Chair on Governance and Transformation of Health Organizations and Systems, Montreal, Canada

**Keywords:** Responsible research and innovation, Health technology, Health systems, Sustainability, Health equity

## Abstract

The scholarship on responsible research and innovation (RRI) aims to align the processes and outcomes of innovation with societal values by involving a broad range of stakeholders from a very early stage. Though this scholarship offers a new lens to consider the challenges new health technologies raise for health systems around the world, there is a need to define the dimensions that specifically characterise responsible innovation in health (RIH). The present article aims to introduce an integrative RIH framework drawing on the RRI literature, the international literature on health systems as well as specific bodies of knowledge that shed light on key dimensions of health innovations. Combining inductive and deductive theory-building strategies and concomitant with the development of a formal tool to assess the responsibility of innovations, we developed a framework that is comprised of nine dimensions organised within five value domains, namely population health, health system, economic, organisational and environmental. RIH provides health and innovation policy-makers with a common framework that supports the development of innovations that can tackle significant system-level challenges, including sustainability and equity.

## Background

There is a steadily growing number of new technologies being introduced in health systems (e.g. wearable devices, robotics, genomics, artificial intelligence, 3D printing, mobile applications, etc.) that raise complex policy challenges “*for all health stakeholders, including policy makers, regulatory authorities, payers, physicians and patients*” [1]. Since the current ways in which new health technologies are being financed, developed and brought to market render health systems increasingly inequitable and unsustainable, it is imperative to develop an integrated policy framework that reconciles the distinct goals of health and innovation policies in order to better articulate the supply of innovation to the demand of health systems [2].

With its critical and reflexive stance towards the purposes and impacts of innovation, the scholarship on responsible research and innovation (RRI) offers a relevant lens to consider the equity and sustainability challenges that new health technologies raise for health systems around the world. This policy-oriented scholarship seeks to align science and innovation processes and outcomes with important societal values, needs and expectations [3]. Yet, there is a need to flesh out the dimensions that are specific to responsible innovation in health (RIH) and are neglected within the more generic and largely decontextualised RRI approach [4].

Henceforth, the aim of this paper is to introduce an integrative RIH policy-oriented framework that is meant to inform the work of public actors who influence the supply of health innovations such as health research funding agencies, public venture capitalists, technology transfer offices and incubators. This framework was developed iteratively by combining deductive and inductive theory-building strategies [5]. Since our ultimate aim is to develop a formal tool to assess the responsibility features characterising innovations, we began by synthesising the key concepts from the RRI literature that were applicable to health innovations and the gaps and specificities a RIH framework needed to address. This was achieved by drawing from lessons learned in the international literature on health systems as well as specific bodies of knowledge that shed light on key dimensions of health innovations, ranging from health technology assessment (HTA) to entrepreneurship.

This article is comprised of three sections. Below, we summarise the concepts and principles of RRI and the gaps RIH needs to bridge. We then present a RIH framework that emphasises nine dimensions organised within five value domains, namely population health, health system, economic, organisational and environmental. Our discussion explains why it is crucial to provide health and innovation policy-makers with a common framework that deliberately fosters the development of innovations that are responsive to system-level challenges and support more equitable and sustainable health services.

### The emergence, concepts and principles of RRI

RRI has grown rapidly over the past decade and has significantly influenced the European research policy landscape, especially through its integration within *Horizon 2020*, the European Union Framework Programme for Research and Innovation for 2014–2020. Aiming to produce better science and take ‘real-world complexities’ into account, RRI offers a broad set of principles and strategies to enable academics and stakeholders to address the seven “*Grand Challenges*” identified by the European Community, ranging from “*smart, green and integrated transport*” to “*food, agriculture, forestry and water*” (https://www.rri-tools.eu/about-rri).

Whilst RRI may be considered as a relatively new line of investigation, the social impact of science and technology has been an area of scientific concern since the late 1940s. Philosophers and sociologists of science and technology as well as ethicists and experts in technology assessment introduced a range of concepts and frameworks that have been refined and extended over the years. In the early 1990s, with the emergence of genetics and genomics, formal research programmes on their ethical, legal and social issues were created. For example, the Ethical, Legal and Social Implications (ELSI) programme was formally established in the United Sates in 1990, whilst the acronym ELSA, standing for Ethical, Legal and Social Aspects, was introduced in the context of the 4th European Union Framework Programme in 1994. During the same period, academic programmes, journals and conferences in science and technology studies were consolidated in Europe, North America and Asia. Such scholarly endeavours, which have been transformed into contemporary approaches, may thus be seen as predecessors of RRI [6].

Nevertheless, the number of publications that explicitly refer to RRI as well as the number of individuals associated to the field have grown rapidly in recent years. A mapping of the RRI landscape showed that, at the end of 2014, 536 persons affiliated to 246 organisations from 89 countries were involved in RRI [7]. For Timmermans [7], the RRI discourse “*really took off in 2009, when the number of persons involved grew from 2 to 28 and has doubled almost every year since*”. During this period, several books [4, 8–11] and 235 articles dealing with the definitions and concepts of RRI [12] were published and the *Journal of Responsible Innovation* was launched by Tailor & Francis. Although it is yet to be indexed, this journal published 129 articles between 2014 and 2017 and its editors include RRI leaders.

Whilst the RRI scholarship encompasses various conceptualisations of RRI and emphasises slightly different aspects, we extracted the key characteristics from the literature to clarify what RRI is, who should be involved and when, what needs to be done, in what ways and to what ends (Table 1). Following this, RRI may be summarised as follows:

i. RRI is a process, an approach, an endeavour, a meta-responsibility, an ideal or a project
ii. that should involve all actors interested in, or affected by research and innovation (R&I) activities
iii. at an early stage and throughout the whole lifecycle of R&I
iv. to anticipate, monitor and assess social, economic and environmental impacts and implications of R&I activities, reflect on the purposes, motivations, assumptions, values, beliefs, uncertainties, risks and dilemmas by engaging diverse stakeholders, including civil society, to dialogue and deliberate, and to react and respond to changing values and circumstances
v. by working together and becoming mutually responsive to each other in an open, inclusive, deliberative and timely way
vi. in order to take the “*effects and potential impacts* [of R&I] *on environment and society*” into account [13], to “*allow a proper embedding of scientific and technological advances in society*” [14, 15], to “*solve a set of moral problems*” [16], to “*create a society in which R&I practices strive towards sustainability, ethically acceptable, and socially desirable outcomes*” [17] and to “*take care of the future*” [18].

**Table 1:** A summary of responsible research and innovation (RRI) according to the literature

What RRI is	- A process [10, 14–17] - An approach [13, 67] - An endeavour [68] - A meta-responsibility [69] - An ideal and a project [19]
Who should be involved	- Societal actors and innovators [14, 15, 70] - Scientists, innovators, business partners, research funders and policy-makers [68] - All stakeholders involved in research and innovation (R&I) practice [17, 67] - Funders, researchers, stakeholders and the public [71] - Large community of people [19] - All parties [12]
When	- Early stage of R&I processes [12, 13] - Any stage of R&I processes [67] - R&I processes as a whole [70] - Throughout the entire innovation’s lifecycle [10]
To do what	- Anticipate risks and benefits, reflect on prevailing conceptions, values and beliefs, engage stakeholders and members of the wider public, and respond to stakeholders, public values and changing circumstances [18] - Describe and analyse potential impacts; reflect on underlying purposes, motivations, what is known and not known, uncertainties, risks, assumptions, questions and dilemmas; open these reflections to broad collective deliberations and use this collective process of reflexivity to set the direction and influence subsequent trajectory and pace of innovation [9] - Shape, maintain, develop, coordinate, and align existing and novel R&I-related processes, actors and responsibilities [69] - Monitor social, economic and environmental performance impacts and anticipate corrective actions [10] - Integrate measures throughout the innovation process [10] - Assess the qualities of the innovation process [72] - Anticipate and discern how R&I can or may benefit society [12] - Anticipate and assess potential implications and societal expectations with regard to R&I [13]
In what ways	- Working together [70] - Becoming mutually responsive to each other [14, 15, 17] - Open, inclusive and in a timely fashion [71]
To what ends	- Allow a proper embedding of scientific and technological advances in society [14, 15] - Better align processes and outcomes with the values, needs and expectations of European society [70] - Take care of the future [18] - Ensure desirable and acceptable research outcomes [69] - Solve a set of moral problems [16] - Create a society in which R&I practices strive towards sustainability, ethically acceptable and socially desirable outcomes [17] - Protect the environment and consider impacts on social and economic dimensions [10] - Take potential impacts on environment and society into account [13] - Promote creativity and opportunities for science and innovation that are socially desirable and undertaken in the public interest [71]

Whilst different emphases may easily coexist in the RRI literature, one object of contention lies with the distinction between a product approach and a process approach [19]. These approaches aim towards the same goal of producing innovations that are responsible, but they propose different ways to reach this goal. On the one hand, a product approach suggests that a normative RRI framework drawing from a set of shared values and norms has to first be developed and then applied in order to define the characteristics a given technology should possess (e.g. being ethically acceptable, sustainable, socially desirable). On the other hand, a process approach frames RRI as an on-going and recursive process that follows a set of normative procedural criteria and is applied to design and develop technologies in a responsible way. Typically, the RRI process requirements include (1) diversity and inclusion; (2) anticipation and reflection; (3) openness and transparency; and (4) responsiveness and adaptation to change [17]. Whilst a large part of the RRI literature is process oriented, some authors underscore the importance of examining both product and process [15, 20]. As we will further develop in the next section, a RIH framework should encompass process and product, but it also needs to characterise the organisation that is supposed to apply such processes to design a product that will be brought to market. Otherwise, such a framework would remain unable to grasp the commercial underpinnings of innovation.

The RRI concept and its general applicability have been criticised. Whilst some authors [14] point out that the RRI definition most used by scholars and policy-makers [15] is problematic because of its emphasis on ‘marketable products’ and its Eurocentric vision of society, others [21] challenge the concept of innovation presented in RRI literature, which is overconcerned with technological innovation, primarily perceived from an economic perspective as “*inherently good*” and which “*presupposes a symmetry between moral agents and moral addressees*”. According to Zwart [6], who assessed the recent shift in the European research funding arena, what differentiates RRI from its predecessor (ELSA) are not the methods or proposed approaches, but rather RRI’s focus on socioeconomic benefits of innovation in order to ensure that “*the EU economy remains internationally competitive and robust*” whilst tackling important societal challenges. In addition, issues were raised concerning the input (e.g. limited engagement of stakeholders in innovation processes because of their conflicting agendas and their differences concerning the content of relevant societal challenges), the throughput (e.g. existing asymmetry of power and information, challenging the practice of mutual responsiveness and collaboration among stakeholders) and the output (e.g. impossibility to anticipate all unintended consequences of an innovation) of RRI processes [21].

Although many RRI publications emphasise the importance of partnerships with industry and private companies in order to achieve sustainability and ethical acceptability of research and innovation outcomes, the level of interest manifested by the business community is low. According to Timmermans [7], only 10% of the persons involved in RRI were affiliated to businesses. Likewise, approximately 8% of the participants attending an important RRI conference held in Brussels, Belgium, in January 2016, came from industry [22]. Suggested explanations for entrepreneurs’ lack of involvement are related to the following aspects: (1) RRI lacks definition and clarity due to the variety of similar concepts and approaches (e.g. social innovation, sustainable innovation, open innovation) [23, 24]; (2) RRI emphasises science and technological development and fails to address important stages of the innovation lifecycle (e.g. commercialisation) [23]; (3) the RRI concept has been implemented mainly in the context of publicly funded research, with little effort to adapt and operationalise it for the business context [24]; and (4) business and industry are mainly seen as areas of application instead of partners in RRI projects [7]. As a result, it is not entirely surprising that current discussions on RRI may not capture the attention of the business community. A limited opportunity for dialogue and a lack of mutual interest may partly explain why financial and commercial dynamics are poorly developed in the RRI literature (exceptions worth mentioning include the contributions of Pavie et al. [10], Hin et al. [25] and Iatridis and Schroeder [26]). This is highly problematic considering that the development of new technologies often calls for massive capital investments and their production requires the expertise, skills and know-how of individuals operating within industry.

To summarise, despite important criticisms, the shared principles that RRI seeks to follow are to (1) address societal needs and challenges; (2) engage a range of stakeholders with the aim of improving decision-making and mutual learning; (3) anticipate potential problems, assess available alternatives and reflect on underlying values, assumptions and beliefs; and (4) provide guidance on ways to act in accordance with the previous principles [27]. Whilst these core principles draw on the bodies of knowledge and experiences of other existing frameworks and approaches developed in the context of research and innovation, what would be specific to RRI is the emphasis on what society morally desires to achieve, on the socioeconomics benefits of innovation and on a closer collaboration with industry. These principles provide important foundations for a RIH framework that needs to address existing gaps in the RRI scholarship. Such a framework has to include the organisations (ranging from for-profit to not-for-profit enterprises) one has to consider when examining the processes and products of RIH as well as the entrepreneurial dynamics shaping the development, production and commercialisation of health technologies. A RIH framework should also strive to be applicable beyond industrialised countries since RRI “*has a global outreach*” in its intentions and aims [7].

## An integrative RIH framework

### Defining what is RIH

To define the scope and specificities of our RIH framework, we applied an iterative approach wherein deductive and inductive analytical strategies alternated. Since one of our research team’s objectives was to develop a formal RIH assessment tool, we sought to build on key concepts from the literature, but we also explored whether these concepts meaningfully captured a range of empirical characteristics. Specifically, we generated a preliminary list of dimensions by drawing from the international health systems literature and bodies of knowledge that are particularly relevant to RIH. These included scholarships that are specific to health (e.g. HTA, ELSIs, determinants of health, health economics, health services research, etc.) and others that are specific to technology-based entrepreneurship (e.g. start-ups, incubators, business models, frugal or ‘bottom of the pyramid’ innovation, etc.).

Using a preliminary set of criteria such as innovativeness, health relevance and subsidiarity, we created an inventory of health innovations that could potentially qualify as responsible by performing a structured social media-based horizon scan supplemented with searches of specialised directories and websites. This exercise generated approximately 100 empirical examples that were extremely useful to circumscribe what RIH may entail in practice. Whilst results of this exercise are described in detail elsewhere (under review), we provide below a brief description of three innovations of our inventory to illustrate some of the principles and dimensions underscored by these examples.

The first innovation is a menstrual cup developed by a social enterprise and distributed free of charge to young women in developing countries through strategic partnerships with local organisations and a ‘buy one, give one’ model. Although its initial cost is higher than that of traditional alternatives, the menstrual cup can be used for up to 10 years, making it a much more affordable option in the long run (95% cheaper), as well as more ecological, since a woman uses up to 12,000 disposable sanitary products throughout her reproductive life. The second innovation refers to 3D-printed prostheses created and printed by a global community of volunteers for those in need of upper limb assistive devices (hands and arms). These prostheses are mechanical, customisable, easy to assemble, printed free of charge from anywhere in the world and delivered directly to the user. Their designs are open-source and shared on a website dedicated to the sharing of digital design files created by its users – more than 100 models are currently available at no cost. The third innovation is a portable nebuliser which is manually activated by a healthcare worker or caregiver and does not use batteries or any other external source of energy. It is easy to use, and its performance is equivalent to that of an electric-powered nebuliser. Although it is not yet available on the market, the projected price of the innovation is less than half of that of an electric nebuliser, whilst the cost of operation and maintenance is estimated to be low.

These examples allowed us to think about the value domains that resonate with different types of health innovations and to better understand the scope of dimensions that a RIH should have, such as the promotion of health equity, frugality and eco-responsibility. They also confirmed the importance of appraising the responsibility of a given innovation within the context where its users are located. For instance, the menstrual cup may favour social insertion of women who live in regions of the world where access to safe, effective sanitary products is a major challenge due to their cost or unavailability, which leads to social stigma and school/work absenteeism; 3D printable, open-source prostheses may benefit children who cannot afford traditional prostheses and need to perform simple tasks such as holding a ball, pressing buttons and turning pages; and the human powered nebuliser may be a proper solution for people who live in remote communities where respiratory health problems (e.g. tuberculosis, chronic obstructive pulmonary disease, asthma and lower respiratory tract infections) are prevalent and access to electricity is limited or non-existent.

In short, the inventory of potential examples of RIH enabled our team to gradually consolidate the framework that is presented below. It also contributed to the further development of the RIH Assessment Tool, which aims to identify whether an innovation may potentially qualify as a RIH and, if so, to assess its responsibility features. In order to evaluate and reach a consensus on the constructs of this Tool (screening criteria, assessment attributes and rating system), we conducted an international Delphi study in 2017 by consulting with experts with complementary perspectives on health innovation – RRI scholars, biomedical engineers, bioethicists and HTA experts. The findings of this study have been summarised and submitted for publication (under review) and were extremely helpful to revise and validate the value domains and dimensions of the framework.

Drawing on the RRI core principles presented earlier and the iterative approach summarised above, we define RIH as follows:

RIH consists of a collaborative endeavour wherein stakeholders are committed to clarify and meet a set of ethical, economic, social and environmental principles, values and requirements when they design, finance, produce, distribute, use and discard sociotechnical solutions to address the needs and challenges of health systems in a sustainable way.

RIH is thus defined as an ambitious and sustained effort that requires collaboration among many diverse stakeholder groups (e.g. investors, technology developers, providers, managers and users of health services, regulators, policy-makers, etc.). Collaboration among these groups is certainly fraught with tensions because dominant settings of research, production and sale are not necessarily aligned with all of the many settings of adoption, adaptation and use of health innovations. Nevertheless, collaboration is essential not only from a normative procedural standpoint, but also because the development of innovations must tap into the complementary (and at times conflicting) expertise, know-how and experience these stakeholders possess as they handle different aspects of health innovations (e.g. financing, design, production, regulation, reimbursement, use, etc.).

The term ‘sociotechnical solutions’ emphasises the notion that the use of a technology, either as simple as a scalpel or as complex as a surgical suite, requires the combined action of an array of individuals and technical components. This term calls our attention to solutions that go beyond what is usually understood as health technologies (e.g. medical devices, drugs, vaccines, medical procedures or information systems) and that address the various factors that determine health across one’s life course (e.g. education, employment, physical environment, etc.).

The ‘principles, values and requirements’ of RIH are considered throughout an innovation’s ‘lifecycle’ (i.e. from the choice of materials to be used to end-of-life disposal), thereby encompassing its characteristics, the processes by which it is being produced, the organisation that develops it as well as the suppliers and distributors that are needed to produce and make it available to end users. As a result, RIH moves beyond a silo approach to more comprehensively support the governance of health innovations across multiple sectors and policy domains.

### The value domains and dimensions of RIH

One important premise of our framework is that a given innovation would be deemed irresponsible – and thus excluded from further consideration – if it were not proven effective and safe and if the organisation bringing it to market were engaged in corporate social irresponsibility, which includes behaviours and corporate actions that are illegal, unsustainable or unethical, leading to negative consequences for individuals and ecosystems [28]. As pointed out by Jones et al. [29], some positions adopted by irresponsible organisations concerning corporate issues and the ways they relate to wider society include, but are not limited to, little precaution on environmental degradation and pollution, unfair treatment of suppliers and customers, and development and launch of new technologies regardless of the harm they may cause.

Table 2 presents the five value domains of the RIH framework which comprise of a total of nine dimensions. The first domain, ‘population health value’, suggests that, although innovation that provides individual health benefits is valuable, RIH should increase our ability to attend to collective needs whilst tackling health inequalities [30]. The second domain, ‘health system value’, draws attention to the extent to which an innovation provides an appropriate response to contemporary challenges of health systems [31]. Third, the ‘economic value’ domain, proposes that RIH must deliver both high-performing products as well as affordable ones in order to support equity and sustainability [10, 32]. Fourth, the ‘organisational value’ domain underscores the business strategies through which an enterprise provides value to users, purchasers and society [33]. Finally, the ‘environmental value’ domain highlights the need to reduce, as much as possible, the negative environmental impacts of health innovations throughout their entire lifecycle [34, 35]. These value domains and dimensions provide health and innovation policy-makers with key elements that may foster the development of innovations responsive to system-level challenges and that support more equitable and sustainable health services. In the following paragraphs, we clarify the nature and importance of each dimension that integrates the RIH framework we propose.

**Table 2:** The value domains and dimensions of responsible innovation in health

Value domain	Dimension
Population health	Health relevance: Does the innovation address a relevant health problem? Ethical, legal and social issues: Was the innovation developed by seeking to mitigate its ethical, legal or social issues? Health equity: In what ways does the innovation promote health equity?
Health system	Inclusiveness: Were the innovation development processes inclusive? Responsiveness: Does the innovation provide a dynamic solution to a health system need or challenge? Level of care: Is the level of care required by the innovation compatible with health system sustainability?
Economic	Frugality: Does the innovation deliver greater value to more people using fewer resources?
Organisational	Business model: Does the organisation that produces the innovation seek to provide more value to users, purchasers and society?
Environmental	Eco-responsibility: Does the innovation limit its negative environmental impacts throughout its lifecycle as much possible?

The population health value domain is comprised of three dimensions (Table 2). The ‘health relevance’ dimension seeks to ascertain the importance of the health needs addressed by an innovation within the overall burden of disease, considering the risk factors and causes of morbidity and mortality that are specific to the region where the intended users are located. For example, some types of cancer, such as bladder, kidney or prostate cancer, constitute an important burden of mortality and morbidity in high-income countries, whereas certain infectious diseases (e.g. HIV/AIDS, meningitis, tuberculosis) represent an important burden in developing nations [36]. Since many new technologies tend to provide incremental benefits when compared to existing alternatives, one delicate issue when reflecting on the level of responsibility of an innovation consists of attributing value to its purpose. Because companies and investors are often reluctant to engage in research and development efforts when the putative markets are small or less affluent [37], innovations that address life-threatening or chronically debilitating rare diseases as well as neglected tropical diseases may provide responsible actions to respond to health needs that would not otherwise be met. This first dimension is thus specific to RIH and brings to the fore a collective responsibility toward health needs.

Acknowledging that “*health care is a moral endeavour*” and that “*the vast potential of technology poses complex moral challenges*” [38], the second dimension draws attention to the ELSIs that underlie the development, distribution and use of health technologies. Although such issues cannot be entirely identified in advance, RIH calls for a careful examination of mitigation strategies that are needed according to the context of use. For instance, an assistive device may exacerbate social stigma associated with disability in certain cultural groups or the default options of a monitoring device may not respect one’s individual right to privacy. The “*social shaping of technology*” perspective suggests that the degree of responsibility of an innovation is influenced, implicitly and explicitly, by multiple values as the “*technologies and the actors that develop and implement them are inherently value-laden*” [39]. Accordingly, one would wish to define, at an early stage, the values embedded in innovations that, for instance, build on artificial intelligence such as wearable devices, robotics or mobile applications, in order to identify the mitigation means they require. This second dimension thus adheres to the RRI principle according to which ELSIs have to be reflexively anticipated, thereby extending the scope of the ethical analyses currently performed in HTA [40].

The third dimension emphasises the importance of ‘health equity’, which is aligned with concerns about the varying “*capability to achieve good health*” among different social groups and recognises that the achievement of health is embedded in “*broader issues of social justice and overall equity*” [41]. Current evidence indicates a strong correlation between healthcare disparities, measured in terms of accessibility, continuity and comprehensiveness of care, and vulnerability factors, such as poverty, ethnic minorities, incarceration, immigration status and underserved areas [42]. In addition to these factors, vulnerability may also be generated or exacerbated by (1) health technologies that are used to legitimise discrimination and social inequalities, as illustrated by the built-in racial bias of the spirometer against black people [43], (2) new forms of healthcare that are simultaneously innovative forms of clinical research (e.g. precision medicine), which increase the number of complex decisions that have to be made by patients and their healthcare providers [44], and (3) healthcare delivery models in which access to services is based on ability to pay rather than equity, imposing financial barriers and contributing to significant and damaging financial loss [45]. From this perspective, an innovation may be seen as responsible if it explicitly addresses the needs of groups who are considered vulnerable “*based on the ways in which they are marginalised, socially excluded, have limited opportunities and income, and suffer abuse, hardship, prejudice and discrimination*” [46]. Such vulnerable groups include, but are not limited to, subsistence farmers, long-term unemployed, informally employed and seasonal/daily workers; people living in deprived urban and rural areas, in poverty and homeless; people with disabilities and mental illnesses; ethnic minority groups, asylum seekers and refugees; single parents, older people and children [46, 47]. The degree of responsibility of an innovation may thus be pondered by examining whether the ability to benefit from it varies according to one’s socioeconomic status, social position or capabilities. This dimension is thus grounded in the health literature and underscores that innovations seeking to benefit population health should also strive to promote health equity.

As Table 2 indicates, the ‘health system value’ domain is comprised of three dimensions, starting with a dimension that examines the degree to which innovation processes and outcomes are ‘inclusive’. For Klaassen et al. [17], opening up science and innovation practices to multiple societal actors is important for “*democratic reasons and also to broaden and diversify the sources of expertise and perspectives*”. To achieve inclusiveness in RIH, one must firstly clarify the particular set of individuals and institutions that should contribute to the design, development and pilot stages of an innovation (e.g. health and social care practitioners and managers, patients and informal caregivers, community and civil society representatives, etc.). Secondly, it is important to define what modalities of engagement should be deployed to capture their expectations, needs and interests, to assess the value and importance of these claims and to handle them in an open way. In other words, RIH should rely on the engagement of a well justified set of stakeholders and the ways in which their inputs will or will not be integrated into the innovation should be clear and explicit. Therefore, this dimension is both process- and product-oriented and draws on a RRI principle which stipulates that, even though stakeholder engagement may differ from one type of innovation to another, the processes and outcomes should be held accountable [12].

Responsiveness, an important RRI principle, recognises that unforeseen consequences may result from innovations and that the context in which they are disseminated may shift in unexpected ways. This principle requires the ability “*to develop an answer* [response] *and react* [respond] *to external developments caused either by other actors or the natural environment*” [48]. Within the RIH health system value domain, the ‘responsiveness’ dimension brings to the fore the value of providing flexible and opportune solutions to existing and emerging system-level challenges. According to a scoping review of the international peer-reviewed literature on health systems, approximately two-thirds of the challenges documented by scholars were related to health service delivery (e.g. access, vertical integration, referral systems), human resources (e.g. staff availability, competency and distribution), and leadership and governance (e.g. strategic policies, horizontal coordination) [31]. This suggests that, although the nature and importance of health system challenges vary across countries, responsible innovations should carefully consider their contribution to, and impact on service delivery, human resources and governance. This dimension thus adapts a RRI principle to the systemic context in which health innovations are deployed, keeping in mind the importance of addressing health system needs and challenges in a sustainable way.

The ‘level of care’ dimension seeks to ascertain the extent to which the innovation is compatible with health system equity and sustainability. As MacDonnell and Darzi [49] suggest, innovations that reduce the labour intensity of care should be encouraged to better manage health spending growth. Following the principle of subsidiarity, the most decentralised unit in the health system (e.g. primary care, non-clinical settings) should be mobilised to provide care when it is possible to do so effectively and safely. By increasing the capacity of general practitioners and community health and social care providers to locally attend to their patients’ needs, responsible innovations may reduce geographical access barriers that are characteristic of rural and remote areas. Similarly, innovations that are aligned with patients’ capacity for self-care may offer responsible solutions if high-quality outcomes are achieved. This dimension is thus anchored in health services research and invites a careful consideration of the requirements in terms of infrastructure and specialisation of new technologies.

Within the economic value domain, the ‘frugality’ dimension emphasises the importance of providing more value to more people using fewer resources [50]. According to Tran and Ravaud [51], frugal innovations in health may result from (1) simplification of existing techniques or technologies (e.g. low-cost bubble continuous positive airway pressure, portable electrocardiogram machine); (2) use of modern technologies to tackle ‘old problems’ (e.g. SMS to improve adherence to antiretroviral therapy, 3D printed prostheses); (3) diversion of existing tools for completely different purposes (e.g. solar-powered autoclave, paper clips in surgery); and (4) use of low-tech approaches to solve local unmet needs (e.g. Kangaroo care for preterm infants, solar disinfection of water). Whilst frugal innovation is considered to be the outcome of low-resource settings, where traditional solutions are too expensive, not available or impossible to use with existing resources, the concept of frugality may also be applied to design and develop low-cost, high-quality technologies. Examples of such technologies are provided by Williams [52], who studied how a non-profit ophthalmic consumables and equipment manufacturing company in India was able to successfully design and develop intraocular lenses and ophthalmic drugs to treat non-communicable eye diseases (cataract, glaucoma, age-related macular degeneration, diabetic retinopathy) for marginalised people. In fact, frugality may address RIH process, product and organisational concerns in conjunction by seeking to substantially reduce the cost of an innovation through focusing on the core functionalities its users require and optimising its performance level (i.e. quality, robustness, accuracy, durability) in view of the intended purpose and context of use [53]. Frugality may thus support RIH by increasing affordability because of optimised innovation production processes and/or lower maintenance needs; usability, which enables reaching out to patients who would not otherwise benefit from the innovation, including remote or resource-poor settings; and fit between the innovation’s performance and its context of use. This dimension is informed by the health economics literature on the pressures new technologies exert on the growth of health expenditures [32, 54], but it goes beyond the provision of affordable health interventions.

Within the organisational value domain, the ‘business model’ dimension, which focuses on “*the rationale of how an organisation creates, delivers, and captures value*” [55], acknowledges the tension between value capture (i.e. economic returns) and value creation (i.e. provision of a high-quality product). This tension increases when the performance of the organisation is measured exclusively in economic terms. Research shows that the business models currently established in the medical device industry, coupled with the rapid growth and high return logic of venture capital, tend to generate technologies that health systems can no longer afford and whose added value may remain marginal from a clinical or population health standpoint [33]. Whilst philanthropic organisations may be able to contribute to RIH, their economic sustainability remains challenging [56]. This is why hybrid organisations such as social purpose businesses, cooperatives and enterprising non-profits that rely on alternative, economically viable business models may better support RIH. For Haigh and Hoffman [57], hybrid organisations differ from traditional businesses in that they adopt explicit goals to foster social and/or environmental change, maintain sustainable and mutually beneficial relationships with suppliers, employees and customers, and interact with competitors and other institutions with the goal of benefitting society as a whole. Whilst traditional commercial enterprises may contribute to economic development and offer valuable innovations, organisations that adopt alternative business models are in a position to provide more value to consumers, users and society. The Aravind Eye Hospital, an ophthalmic institution that provides a large proportion of its services (diagnostic examination, ophthalmic surgery and postoperative care) to low-income people in southern India, is a well-known example of a healthcare organisation that adopts an alternative, economically viable business model to deliver high-quality, low cost ophthalmological care to those who have little or no access to major eye care facilities due to geographical and financial barriers [58, 59]. Hence, this dimension draws on the social entrepreneurship scholarship and bridges a gap in the RRI literature.

Finally, within the environmental value domain, the ‘eco-responsibility’ dimension acknowledges the importance of healthcare’s carbon footprint and emphasises the environmental impacts of health innovations throughout their lifecycle [35]. Activities inside and around hospitals tend to consume a lot of energy and raw resources and to produce a range of hazardous materials that may be dangerous, infectious, toxic or radioactive. For instance, activities that have negative environmental impacts include discarded materials and equipment, expired or unused pharmaceutical products, drugs and vaccines, and chemicals generated through disinfecting procedures or cleaning processes. The negative impacts on human health of hazardous material (10–25% of healthcare waste) are numerous, including infectious diseases due to exposure to contaminated waste, intoxication, injuries and poisoning caused by chemical and pharmaceutical waste, and health problems attributable to genotoxic and radioactive waste [60]. Efforts to reduce current negative environmental impacts of health-related activities may thus prove responsible as would the development of ‘green’ technologies [61]. For instance, a new product may be designed so as to be free of substances such as latex, heavy metals or chemicals that are of major public health concern or harmful and toxic to ecosystems. It can be made of recycled or renewable content materials and be designed to be easily recycled, reused, remanufactured, composted or biologically degraded when it reaches its end of life. Compliance with environmental regulations and green certifications as well as efficient energy consumption during its production and/or utilisation may also reflect environmental responsibility. Hence, this dimension draws on a RRI principle and is aligned with a planetary health research priority [62].

## Discussion

### Contribution to health innovation policy

**Fig. 1:**
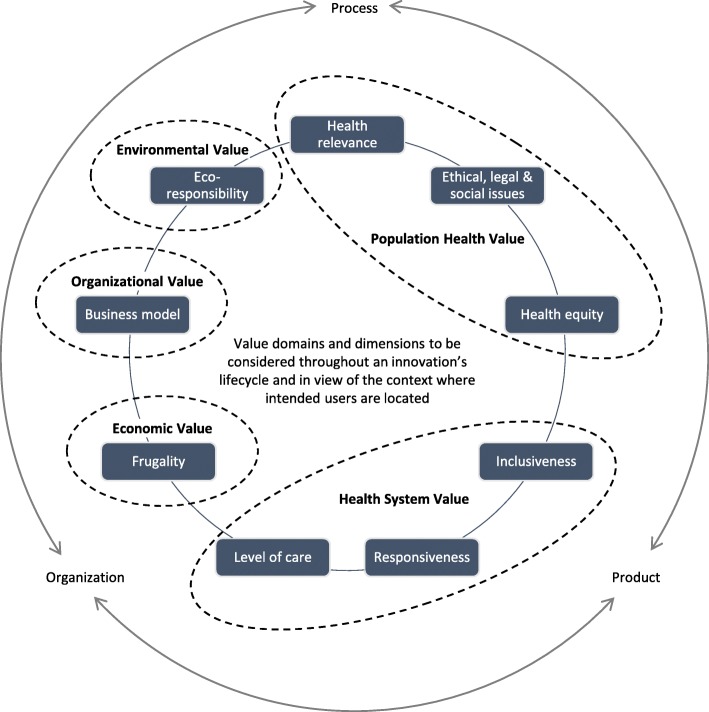
Responsible innovation in health framework

Building on core RRI principles whilst seeking to address problematic gaps, we introduced a RIH framework that adopts a global perspective on health systems, adds ‘organisation’ to the process and product components of innovation, and formally acknowledges what the entrepreneurial endeavour of innovation in health embodies. Figure 1 summarises the nine dimensions of this integrative framework.

We believe that this framework can inform the work of public institutions that directly influence the supply of health innovations, such as health research funding agencies, public venture capitalists, technology transfer offices and incubators, as well as health policy-makers who influence the demand (e.g. through procurement policies, coverage and reimbursement decisions, HTA programmes, etc.). Firstly, it brings to the fore the equity and sustainability challenges that are raised around the world by new health technologies and articulates an integrated set of issues that have to be considered throughout health innovation development processes in order to find solutions that benefit health systems in a responsible way. Whilst responsibility is not a new notion in the health sector, it has been formalised through ethical codes of conduct for healthcare providers and market access regulations regarding the safety and effectiveness of new medical devices and drugs, which come into play in the context of adoption and use, that is, when these innovations can hardly be modified [63]. None of these formal mechanisms provide guidance on ways to develop innovations of greater societal value in terms of health system sustainability and equity. Hence, our framework provides a holistic, yet detailed set of dimensions through which both health and innovation policy-makers can apprehend the context of creation of RIH.

Secondly, the RIH framework responds to the crucial need to provide health and innovation policy-makers with a common framework that deliberately supports the development of innovations that are responsive to system-level challenges and can improve the overall governance of health innovations within health systems. Whilst cost-effectiveness and budget impact analyses, which are typically performed once an innovation is fully developed and ready to hit the market, may inform reimbursement decisions by comparing its costs and consequences to other interventions, many high-cost and poorly performant technologies continue to be developed by manufacturers and adopted in healthcare practice, placing financial pressure on governments, employers and patients [1, 31]. Our framework includes an economic value domain but adopts a system-level perspective that focuses the attention on what the impacts of a given health innovation may look like ‘upstream’, that is before investors and entrepreneurs commit themselves to the development of a particular solution. Whilst we agree that stakeholder involvement in innovation processes does not necessarily guarantee responsible outputs [21], several shortcomings and sources of failure can be anticipated before it gets too late to realign an innovative trajectory, an issue that matters to investors and entrepreneurs as well. In other words, the RIH framework enables multiple stakeholders to engage into productive discussions at an early stage.

### Strengths and limitations of the framework

Whilst the domains and dimensions of our RIH framework were developed by drawing from several bodies of knowledge, its key limitations should be highlighted. First, we cannot ignore the criticism directed at RRI [6, 14, 21]. As stressed by Blok and Lemmens [21], whilst RRI “*presupposes symmetry between moral agents and moral addressees*”, in practice, conflicting agendas, different understandings of what important societal challenges are, and asymmetries of power and information constrain stakeholder engagement, thereby undermining the mutual responsiveness that RRI calls for. This criticism is sound and partly explains why we developed the business model dimension – innovations cannot be understood without paying attention to their commercial underpinnings, which indeed affect stakeholders’ perspectives, motivations and contributions. Our RIH framework supports an open, critical reflection on the ramifications of these asymmetries. Eventually, by generating evidence on process–product–organisation configurations that perform poorly in the five value domains of our framework, researchers will be able to define more clearly when and how private organisations fail to provide solutions that are responsive to the needs and challenges of health systems.

One may question why the framework is comprised of these five value domains and not others. For instance, gender is an important issue in the RRI scholarship and could have been integrated as a distinct dimension. We rather believe that gender should be addressed across several value domains since it is relevant to many dimensions. For instance, gendered assumptions may affect how vulnerable groups’ needs are defined and prioritised (health equity), the expected social roles underlying informal caregiving (ELSIs) and who is considered a relevant contributor to innovation development processes (inclusiveness). The same reasoning applies to other important aspects that are used to discriminate social groups, such as ethnicity and socioeconomic status. Consequently, to strike a balance between parsimony and specificity, we limited the number of value domains, but formulated dimensions that can account for a broad range of responsibility issues. In addition to this flexibility, the notion that responsibility is intimately linked to the context of use should be kept in mind when applying the framework.

Finally, a perplexing limitation lies with the application of RRI principles to other contexts, particularly developing nations. For Macnaghten et al. [64], RRI runs the risk of “*intellectual neo-colonisation*” if its application to the developing world reproduces or reinforces problematic relations. They thus suggest finding appropriate ways to engage RRI in local contexts, cultures and practices, and support a dialogue with actors and institutions in the global South. Similarly, Vasen [65] highlights the problem of uncritically transferring Western conceptual frameworks to countries where available resources are limited, and R&I activities are seen as means to achieve higher socioeconomic development. Focusing his analysis on Latin America, he argues that RRI should encompass issues pertaining to mature technologies, adopt a critical perspective that acknowledges the globalisation of the economy, and flesh out theoretical linkages between RRI and social inclusion, inequality and social justice. Although our RIH framework may not be straightforwardly adapted to certain countries, it was developed having in mind regions where established technologies are too expensive, not available or impossible to use with existing resources, as well as examples of sociotechnical solutions that highlight the importance of the health relevance, health equity and frugality dimensions. In doing so, it can contribute to the adoption of more appropriate models of technoscientific development [66] and the emergence of technologies that address structural inequalities “*by meeting the needs or wants of unknown and known non-users by intervening in multiple stages of the technology’s lifecycle*” [52].

## Conclusions

The concept of RIH sheds light on ways to design, finance, produce, distribute and use innovations of greater societal value. It refers to innovations that increase our ability to attend to collective needs whilst tackling health inequalities, provide an appropriate response to contemporary challenges of health systems, deliver high-performing and affordable products, are aligned with business strategies through which an enterprise provides more value to users, purchasers and society, and reduce as much as possible the negative environmental impacts of health innovations throughout their entire lifecycle.

Whilst the integrative RIH policy-oriented framework we presented may certainly benefit from further refinements, it provides an integrated set of dimensions through which health and innovation policy-makers can envision what types of innovations health systems need and how they should be produced and brought to market in order to support equitable and sustainable health systems around the world.

## Abbreviations

ELSA: ethical, legal and social aspects
ELSI: ethical, legal and social implications
HTA: health technology assessment
R&I: research and innovation
RIH: responsible innovation in health
RRI: responsible research and innovation
